# A Krüppel-like factor establishes cellular heterogeneity during schistosome tegumental maintenance

**DOI:** 10.1371/journal.ppat.1013002

**Published:** 2025-03-28

**Authors:** Lu Zhao, George R. Wendt, James. J. Collins III

**Affiliations:** 1 Department of Pharmacology, University of Texas Southwestern Medical Center, Dallas, Texas, United States of America; 2 Howard Hughes Medical Institute, UT Southwestern Medical Center, Dallas, Texas, United States of America; Duke-National University of Singapore, SINGAPORE

## Abstract

Schistosomes are blood dwelling parasitic flatworms that can survive in the circulation of their human hosts for decades. These parasites possess a unique syncytial skin-like surface tissue known as the tegument that is thought to be uniquely adapted for survival in the blood by mediating evasion of host defenses. Previous studies have shown that cell bodies within the tegumental syncytium are turned over and perpetually replaced by new tegumental cells derived from a pool of somatic stem cells called neoblasts. Thus, neoblast-driven tegumental homeostasis has been suggested to be a key part of the parasite’s strategy for long-term survival in the blood. However, the comprehensive set of molecular programs that control the specification of tegumental cells are not defined. To better understand these programs, we characterized a homolog of a Krüppel-like factor 4 (*klf4*) transcription factor that was identified in previous single-cell RNA sequencing (scRNAseq) studies to be expressed in a putative tegument related lineage (TRL) of *Schistosoma mansoni.* Here, using a combination of RNAi, coupled with scRNAseq and bulk RNAseq approaches, we show that *klf4* is essential for the maintenance of an entire TRL. Loss of this *klf4*^+^ TRL resulted in loss of a subpopulation of molecularly unique tegument cells, without altering the total number of mature tegumental cells. Thus, *klf4* is critical for regulating the balance between different cell populations within the tegumental progenitor pool and thereby influences tegumental production dynamics and the fine-tuning of the molecular identity of the mature tegument. Understanding the functions of distinct populations of cells within the tegumental syncytium is expected to provide insights into parasite defense mechanisms and new avenues for combatting the disease these worms cause.

## Author summary

Schistosomiasis is a disease affecting over 250 million people worldwide, caused by schistosomes, which can live in the blood vessels of their hosts for years. The long-term survival of these parasites relies on a special skin-like syncytium called the tegument, which is constantly renewed by a pool of somatic stem cells that differentiate and fuse with the tegumental syncytium. However, a deep understanding of the molecular control of tegumental specification and differentiation is lacking. Previous studies have speculated that there are two molecularly distinct cell lineages that contribute to the tegument of *Schistosoma mansoni*. Here, we describe a homolog of the Krüppel-like transcription factor 4 (*klf4*) that is expressed in one of these two cell lineages. We observe that RNAi of *klf4* results in complete loss of the entire *klf4*^*+*^ lineage as well as a subset of molecularly unique tegumental cells. Remarkably, we observed a compensatory flux of cells via the *klf4*^-^ lineage and thus no change in the total number of mature tegumental cells was observed. Our findings suggest that *klf4* is not required for the maintenance of tegumental cell numbers but for specifying a molecularly unique collection of tegumental progenitors. Thus, loss of *klf4* is anticipated to alter the molecular makeup of the mature tegument, in turn compromising tegumental function *in vivo*.

## Introduction

Schistosomes are parasitic flatworms that infect more than 250 million people around the world causing tens of thousands of deaths and devastating morbidity in the developing world [1]. These parasites are capable of surviving inside the blood of their human host for decades in the face of the host’s immune system and the tremendous physical challenges imposed by living in the circulation [2–4]. The parasite’s syncytial skin-like surface coat, called the tegument, is thought to be essential for not just surviving but thriving in this hostile environment [5–7]. As the interface between the parasite and the host, the tegument is involved in acquiring nutrients to support worm growth [8,9] and in evading host immune responses [7].

The tegument of adult schistosomes has a complicated architecture, comprised of thousands of nucleated cell bodies that connect to the outer tegument via cellular projections extending through the body wall muscles [10,11]. Interestingly, previous studies have shown that schistosome tegumental cell bodies are subject to perpetual turnover and replacement [11,12]. This replacement of tegumental cell bodies is driven by populations of somatic stem cells, called neoblasts, that specify a pool of short-lived *tsp-2*^+^ tegument progenitor cells that express an mRNA encoding TSP-2 [12], a well-characterized anti-schistosome vaccine candidate that is highly expressed in the tegument [13]. These *tsp-2*^+^ progenitors continuously migrate through the worm to fuse with the tegumental cell bodies, ensuring constant turnover [11]. Though the tegument is a continuous syncytium that covers the entire surface of the schistosome, meaning all tegument cells ostensibly share a common cytoplasm, we previously uncovered signs of molecular heterogeneity in both the *tsp-2*^+^ tegument progenitor pool and the mature tegumental cells [11]. Subsequent single-cell RNA sequencing (scRNAseq) studies went on to reveal two molecularly distinct *tsp-2*^+^ tegument-related lineages (TRLs) [14], one that expressed the tegument marker *sm13* [15], while the other expressed an Endoglycoceramidase (*egc*), the micro-exon gene *meg-1,* and the zinc finger protein *zfp-1-1* [11]. Thus, important questions remain about the purpose of these two TRLs. In particular, whether both TRLs represent true tegumental cell progenitor populations and, if so, whether they play a role in establishing the molecular heterogeneity within the tegument.

Here, we examine a Krüppel-like factor 4 (KLF4) homolog whose expression was specifically enriched in the *egc*^+^*/meg-1*^+^*/zfp-1-1*^+^ TRL. Knockdown of *klf4* resulted in ablation of the entire *klf4*^+^ TRL and resulted in a concomitant loss of a molecularly-unique subpopulation of tegument cells. Surprisingly, loss of the *klf4*^+^ TRL had no effect on the overall maintenance of tegument cell numbers. Instead, loss of the *klf4*^+^ TRL was accompanied by a compensatory increase in the flux through the intact *sm13*^+^ TRL. This suggests that parasites rely upon the balance between two different tegument progenitor lineages to fine-tune the molecular identity of the mature tegument.

## Results

### A Krüppel-like factor (*klf4)* homolog is expressed in a TRL

**Fig 1:**
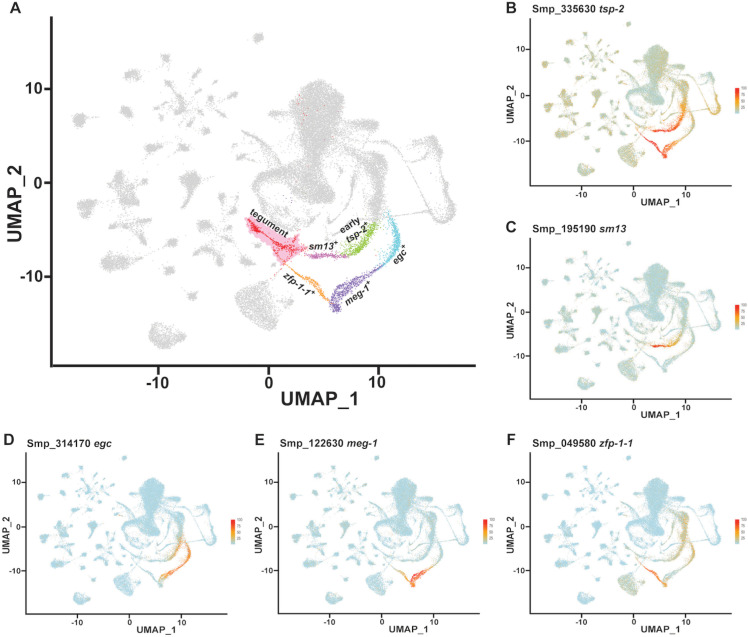
scRNAseq atlas of the adult schistosome reveals two tegument related lineages (TRLs). (A) Uniform Manifold Approximation and Projection (UMAP) showing a scRNAseq atlas of the adult schistosome revealing two lineages predicted to produce tegumental cells. One lineage is characterized by the expression of *tsp-2* and *sm13*, while the other lineage expresses *tsp-2* along with *egc*, *meg-1* and *zfp-1-1*. Different highlighted colors represent distinct TRL^+^ cell populations. (B-F) UMAP expression of *tsp-2* (B), *sm13* (C), *egc* (D), *meg-1* (E) and *zfp-1-1* (F) in TRLs.

**Fig 2:**
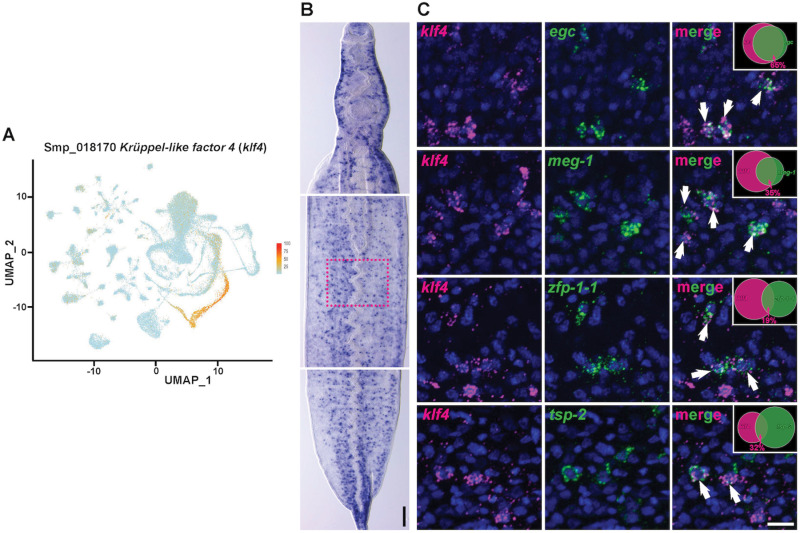
*klf4* is expressed in the *egc*^*+*^*/meg-1*^*+*^*/zfp-1-1*^*+*^ TRL. (A) UMAP showing *klf4* expression in *egc*^+^/*meg-1*^+^/*zfp-1-1*^+^ TRL. (B) Colorimetric whole *in situ* hybridization (WISH) showing expression pattern of *klf4*. Scale bar, 100 µm; anterior towards the top. (C) Double fluorescence *in situ* hybridization (FISH) showing expression of *klf4* relative to the *egc*^+^, *meg-1*^+^, *zfp-1-1*^+^ and *tsp-2*^+^ cells (co-expression indicated by arrows). 65%, 35%, 19% and 32% of *klf4*^+^ cells are *egc*^+^ (81/125 cells, n=3 parasites), *meg-1*^+^ (88/255 cells, n=4 parasites), *zfp-1-1*^+^ (59/319 cells, n=6 parasites) and *tsp-2*^+^ (70/217 cells, n=6 parasites), respectively, as indicated in the Venn diagram in the upper right. The approximate selected region for cell quantification is indicated by the dotted rectangle in panel (B). Scale bar, 10 µm.

Our previously published scRNAseq atlas of adult worms [14], identified TRLs predicted to produce tegumental cells (Fig 1A and S1A Fig) based on their expression of the mRNAs that encode the tegumental protein TSP-2 [13] (Fig 1B). One TRL was characterized by the expression of *tsp-2* (Smp_335630) and *sm13* (Smp_195190) (Fig 1A–1C), whereas the other TRL expressed *tsp-2* along with *Endoglycoceramidase* (*egc,* Smp_314170), *meg-1* (Smp_122630), and *zfp-1-1* [11] (Fig 1D–1F and S1B Fig). The successive expression of *egc*, *meg-1* and *zfp-1-1* on the UMAP plot suggested that these genes could be progressively activated during the differentiation of neoblasts. To evaluate the lineage relationships between *egc*, *meg-1* and *zfp-1-1*, we performed EdU pulse-chase experiments. Consistent with our UMAP plot and the model that *egc*, *meg-1* and *zfp-1-1* exist within a cellular lineage, we observed EdU was first chased into *egc*^+^ cells, then *meg-1*^+^ cells, followed by *zfp-1-1*^+^ cells (S1C-P Fig). To explore the functions of these lineages, we explored our scRNAseq data and found that a homolog of a Krüppel-like factor 4 (KLF4) transcription factor-encoding gene (Smp_018170*, klf4* for brevity) (S2A Fig) was highly expressed in the *egc*^+^/*meg-1*^*+*^/*zfp-1-1*^*+*^ TRL (Fig 2A). Whole mount *in situ* hybridization (WISH) showed that this gene is expressed in discrete cells throughout the body (Fig 2B) and double fluorescence *in situ* hybridization (FISH) confirmed the expression of *klf4* in *egc*^+^, *meg-1*^+^ and *zfp-1-1*^+^ cells (Fig 2C and S2B Fig). Additionally, *klf4* was co-expressed with the tegument progenitor marker *tsp-2* (Fig 2C bottom), but not in cells expressing *sm13* that mark the other TRL (S2C Fig). These data suggest *klf4* is abundantly and specifically expressed in one of the two putative TRLs.

### *klf4* is required for the maintenance of the *egc* ^+^/*meg-1* ^+^/*zfp-1-1* ^+^ TRL

**Fig 3:**
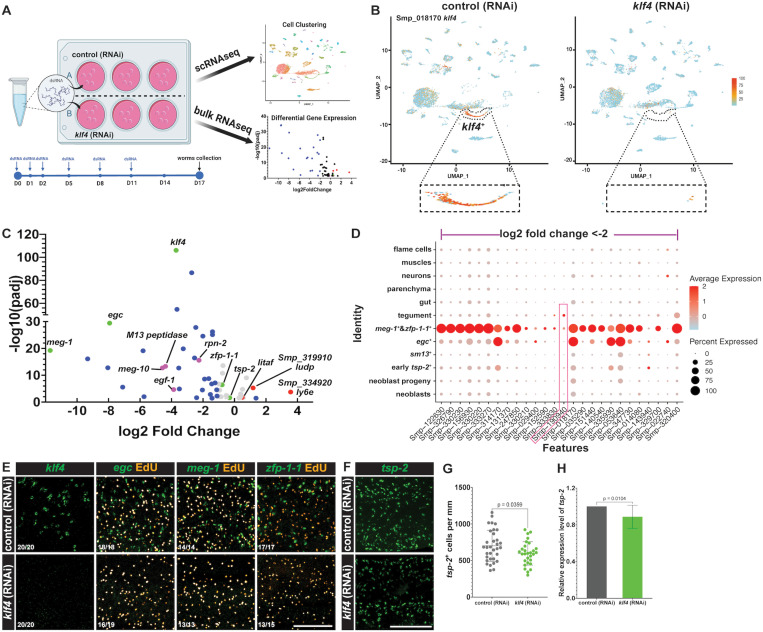
*klf4* is required for the maintenance of the *egc*^*+*^*/meg-1*^*+*^*/zfp-1-1*^*+*^ TRL. (A) Graphic depicting the workflow for exploring function of *klf4* by scRNAseq and bulk RNAseq transcriptional profiling analysis. Adult male schistosome worms were treated with dsRNA targeting *klf4 in vitro* at Days 0, 1, 2, 5, 8 and 11. After 17 days, both control (RNAi) and *klf4* (RNAi) worms were collected for scRNAseq and bulk RNAseq analyses. (B) scRNAseq analysis shows *klf4* RNAi resulted in a loss of the *egc*^*+*^/*meg-1*^+^/*zfp-1-1*^+^ TRL. (C) Volcano plot depicting bulk RNAseq analysis. This analysis identified 67 differentially expressed genes (*P*adj<0.05, 55 down- and 12 up-regulated genes) following knockdown of *klf4*. Grey dots represent genes with a log2 fold change (log2FC) between -1 and 1. Blue dots represent log2FC<-1 or >1. Green dots indicate marker genes expressed in the *egc*^+^/*meg-1*^+^/*zfp-1-1*^+^ TRL. Magenta dots indicate down-regulated genes validated to be expressed in the tegument and *zfp-1-1*^+^ cells. Red dots indicate up-regulated genes validated to be expressed in *sm13*^+^ cells. (D) A dot-plot summarizing the expression of the bulk RNAseq down-regulated differentially expressed genes (DEGs, log2 fold change<-2, *P*adj<0.05) in clusters from the *klf4* RNAi scRNAseq profile in panel 3B. Cluster populations are on the vertical axis and gene IDs are on the horizontal axis. Expression levels are colored by gene expression (blue = low, red = high). Percentage of cells in the cluster expressing the gene is indicated by the size of the circle. (E) FISH results validating the loss of *klf4*^+^, *egc*^+^, *meg-1*^+^ and a substantial reduction in *zfp-1-1*^+^ cells following *klf4* RNAi. We noted no changes in the number of EdU^+^ proliferative cells. Scale bar, 100 µm. Numbers at bottom left represent the fraction of parasites displaying the observed phenotype. (F) FISH depicting a modest decrease in *tsp-2*^+^ cell number following *klf4* RNAi. Scale bar, 100 µm. (G) Quantification of the number of *tsp-2*^+^ cells per mm of worm. Control (RNAi) n= 33, *klf4* (RNAi) n=29*.* (H) qPCR quantification of expression of *tsp-2* following *klf4* RNAi. n=12 experiments. Data are presented as mean ± standard deviation (mean ± SD). An unpaired t-test and a paired t-test were performed in Panel G and H, respectively.

To further investigate the function of *klf4,* we performed RNAi of *klf4* on adult male *S. mansoni*, then performed transcriptional profiling using both scRNAseq and bulk RNAseq to characterize cellular and molecular changes associated with *klf4* loss of function (Fig 3A). Our scRNAseq analysis showed that *klf4* RNAi resulted in a complete loss of the *egc*^+^/*meg-1*^+^/*zfp-1-1*^+^ TRL (Fig 3B and S3A–3E Fig; S1 Dataset), and bulk RNAseq analysis revealed 67 differentially expressed genes (DEGs), of which 55 are down-regulated genes, following *klf4* RNAi (Fig 3C, S2 Dataset). Mapping these DEGs onto the scRNAseq atlas, depicted in Fig 3B, demonstrated that 71% (39/55) of the down-regulated genes were enriched in the *egc*^+^/*meg-1*^+^/*zfp-1-1*^+^ TRL (Figs 3D and S3F). To confirm these data, we performed FISH and qPCR to evaluate gene expression changes in worms subjected to *klf4* RNAi. The results confirmed a complete loss of *klf4*^+^, *egc*^+^ and *meg-1*^+^ cells, as well as a substantial reduction in *zfp-1-1*^+^ cells (Fig 3E and S3G Fig), without any significant changes in the number of proliferative EdU^+^ neoblasts in these worms (Fig 3E and S3H Fig). Additionally, *klf4* RNAi also led to a ~15% reduction in *tsp-2* transcript levels (Figs 3F–3H), consistent with our bulk RNAseq analysis (Fig 3C green dot, S2 Dataset). These results indicate that *klf4* is essential for the maintenance of the *egc*^+^/*meg-1*^+^/*zfp-1-1*^+^ TRL.

### The *egc* ^+^/*meg-1* ^+^/*zfp-1-1* ^+^ TRL is required for producing a specific tegumental subpopulation

**Fig 4:**
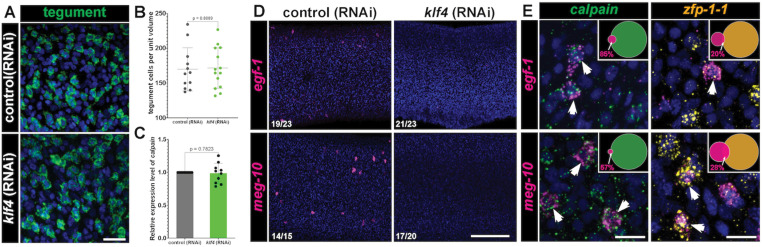
The *egc* ^**+**^**/*meg-1***^**+**^**/*zfp-1-1***^**+**^ **TRL is required for producing a specific tegumental subpopulation.** (A) FISH monitoring the expression for a cocktail of tegumental markers (*calpain* Smp_214190*, npp-5* Smp_153390*, annexin* Smp_077720 and *gtp-4* Smp_105410) in control (RNAi) and *klf4* (RNAi) worms. Scale bar: 50 µm. (B) Quantification of tegumental cell density. Control (RNAi) n= 12, *klf4* (RNAi) n=14. (C) qPCR detection of expression of the tegumental marker *calpain* [11]*.* n=10 experiments. (D) FISH results confirm loss of *egf-1*^+^ and *meg-10*^+^ (highlighted with magenta dots in (Fig 3C)) following *klf4* RNAi. Numbers at bottom left represent the fraction of parasites displaying the observed phenotype. (E) Double FISH showing expression of *egf-1* and *meg-10* relative to the *calpain*^+^ and *zfp-1-1*^+^ cells, respectively (indicated in arrows). The Venn diagram in the upper right shows the percentage of *egf-1*^*+*^ cells that are *calpain*^+^ cells (201/235 cells, n=17 parasites) or *zfp-1-1*^+^ (12/60 cells n=10 parasites) and the percentage of *meg-10*^*+*^ cells that are *calpain*^+^ or *zfp-1-1*^+^ (127/221 cells are *calpain*^+^, n=13 parasites; 66/233 cells are *zfp-1-1*^+^, n=10 parasites). Scale bars, 100 µm. Data are presented as mean ± SD. An unpaired t-test and a paired t-test were performed in Panel B and C, respectively.

Given the profound effects of *klf4* RNAi treatment on the maintenance of the *egc*^+^/*meg-1*^+^/*zfp-1-1*^+^ TRL, we evaluated the effects of *klf4* depletion on tegumental maintenance. Surprisingly, despite ablating an entire TRL, knockdown of *klf4* resulted in no change in the total number of tegument cells (Figs 4A and 4B) and likewise had no effect on the transcript levels of definitive tegumental marker *calpain* (Fig 4C). However, our bulk RNAseq studies found that 16% of the down-regulated DEGs (9/55) were expressed in the tegumental cell population identified by scRNAseq (S3F Fig). Among them was an *EGF-like domain-containing protein* (Smp_190940*, egf-1*) that was highly downregulated following *klf4* RNAi treatment (Figs 3C and 3D, highlighted in pink rectangle). We observed that *klf4* RNAi resulted in complete loss of cell populations expressing *egf-1* (Fig 4D and S4A Fig). Among these *egf-1*^+^ cells, ~80% expressed the tegumental marker *calpain* while another ~20% expressed *zfp-1-1 *(Fig 4E top). These data are consistent with *egf-1* being expressed in a transition state between *zfp-1-1*^*+*^ cells differentiating to definitive *calpain*^*+*^ tegument cells. Similarly, following *klf4* RNAi, we observed a loss of the *meg-10*^*+*^ (Smp_152590) cell population (Fig 4D bottom). Similar to *egf-1*, the majority (~60%) of *meg-10*^+^ cells co-expressed *calpain*, suggesting that they are definitive tegument cells (Fig 4E bottom). We noted similar expression patterns with genes encoding an *M13 peptidase* homolog (Smp_333830) and a *Ribophorin-2* homolog (*rpn-2,* Smp_329700) (S4B-D Fig). Taken together, these data suggest that *klf4* is essential for the maintenance of a subset of tegumental cells that express *egf-1 and meg-10* but is dispensable for the overall maintenance of tegumental cell numbers.

### Knockdown of *klf4* alters the heterogeneity of the *sm13* ^+^ TRL

**Fig 5:**
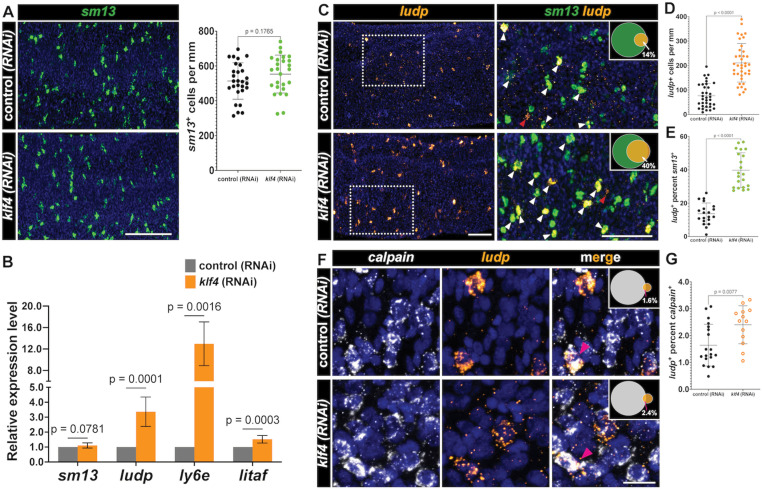
Knockdown of *klf4* alters the heterogeneity of the *sm13* ^**+**^ **TRL.** (A) FISH result showing *klf4* RNAi causes no significant effect on the number of cells expressing *sm13* (left), quantification of the number of *sm13*^+^ cells per mm of worm (right). Control (RNAi) n= 28, *klf4* (RNAi) n=26. (B) qPCR detection of the expression of *sm13* (n=10 experiments) and up-regulated DEGs including *ludp* (Smp_319910) (n=10 experiments)*, ly6e* (Smp_334920) (n=6 experiments) *and litaf* (Smp_333330) (n=10 experiments) following *klf4* RNAi. (C) FISH results showing a significant increase in the number of *ludp*^+^ cells following *klf4* RNAi (left); Double FISH (inset region indicated by the dotted rectangle from the left panel) showing expression of *sm13* relative to the *ludp*^+^ cells (right); the Venn diagram in upper right shows the percentage of *sm13*^*+*^ cells expressing *ludp*. White arrows indicate the *sm13*^+^*ludp*^+^ cells, red arrows indicate the *sm13*^-^*ludp*^+^ cells. Scale bar, 100 µm. (D) Quantification of the number of *ludp*^+^ cells per mm of worm. Control (RNAi) n=34, *klf4* (RNAi) n=38. (E) Quantification of percentage of *sm13*^*+*^ cells expressing *ludp*. Control (RNAi) n=20, *klf4* (RNAi) n=21. (F) Double FISH showing expression of *calpain* relative to the *ludp*^+^ cells, and the Venn diagram in upper right shows the percentage of *calpain*^*+*^ cells expressing *ludp*. Control (RNAi) n=19, *klf4* (RNAi) n=13. Scale bar, 10 µm. (G) Quantification of percentage of *calpain*^*+*^ cells expressing *ludp*. Scale bar, 10 µm. Data are presented as mean ± SD. Welch’s t-tests were performed in Panel A, D, E and G, and multiple paired t-tests were performed in Panel B.

**Fig 6:**
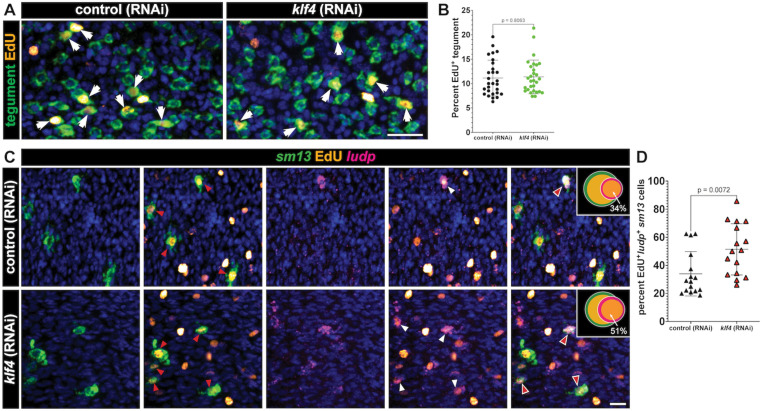
Knockdown of *klf4* increases the production of *ludp* ^***+***^***sm13***^***+***^ ***cells.*** (A) FISH for tegumental markers with EdU detection in control (RNAi) and *klf4* (RNAi) worms at day seven (D7) following an EdU pulse. Arrows represent EdU^+^ tegumental cells. (B) Quantification of the percentage of tegumental cells that are EdU^+^ following a 7-day chase period. Control (RNAi) n=28, *klf4* (RNAi) n=29. (C) Double FISH for *sm13* and *ludp* with EdU detection in control (RNAi) and *klf4* (RNAi) worms at D7 following an EdU pulse. Red arrows represent EdU^+^*sm13*^+^ cells, white arrows represent EdU^+^*ludp*^+^ cells and red arrows with white outline represent EdU^+^*ludp*^*+*^*sm13* cells. (D) Quantification of percentage of *ludp*^*+*^*sm13*^*+*^ cells that are EdU^+^ following a 7-day chase period following *klf4* RNAi. Control (RNAi) n=16, *klf4* (RNAi) n=16. Scale bar, 10 µm. Data are presented as mean ± SD. An unpaired t-test and a Welch’s t-test were performed in Panel B and D, respectively.

Since loss of *klf4* resulted in loss of *egf-1*^*+*^ and *meg-10*^*+*^ tegumental cells without compromising total tegumental cell number, we reasoned that there might be a compensatory increase in the rate at which cells in the *sm13*^*+*^ TRL contribute to the tegument. If this were the case, we would anticipate that we would observe an increase in the expression of markers associated with the opposing *sm13*^+^ TRL. Consistent with this prediction, our bulk RNAseq analysis found that of the 12 up-regulated DEGs following *klf4* RNAi (Fig 3C, S2 Dataset), 8 of these were expressed in the *sm13*^+^ TRL (S5A Fig). Though *klf4* RNAi did not significantly change the number of *sm13*^*+*^ cells or mRNA levels (Figs 5A and 5B), it led to a 3-fold increase in the expression of *ludp* (Smp_319910) (Fig 5B), which encodes a LY6/uPAR domain containing protein, that is predicted to be expressed in *sm13*^+^ cells by scRNAseq (S5A Fig). This increase in *ludp* mRNA levels aligns with an observed 2.6-fold increase in the number of *ludp*^*+*^ cells in *klf4* RNAi worms relative to controls (Figs 5C left and 5D). One potential model to explain this increase in *ludp*^+^ cells is that these cells are in a transition state between *sm13*^*+*^ progenitors and *calpain*^*+*^ definitive tegumental cells and loss of *klf4* results in an increase in the flux of cells via this lineage. To test this model, we examined the expression of *ludp* relative to *sm13* and *calpain* in *klf4* (RNAi) parasites. We observed that 14% of *sm13*^+^ cells are *ludp*^+^ in control (RNAi) parasites, and that the proportion of *sm13*^+^*ludp*^+^ double positive cells jumped to 40% cells in *klf4* (RNAi) worms (Figs 5C right and 5E). We also found that *ludp* was indeed expressed in a small fraction of *calpain*^+^ cells (1.6%) and *klf4* RNAi resulted in a significant increase in the number of *ludp*^+^*calpain*^+^ double positive cells (1.6% to 2.4%) (Figs 5F and 5G). These results were mirrored with two other genes (*Lymphocyte antigen 6E-like protein* (*ly6e*, Smp_334920) [16, 17] and *litaf domain containing protein* (*litaf,* Smp_333330) in terms of increases in the number of double positive cells in the *sm13*^+^ TRL (S5B–S5D and S5G-I Figs); however, no increases in the number of double positive cells were noted in the definitive tegumental cell compartment (S5E, S5F and S5J, S5K Figs). These data suggest the possibility that loss of *klf4* prohibits neoblasts from committing to the *egc*^+^/*meg-1*^+^/*zfp-1-1*^+^ TRL, in turn resulting in more *ludp*^+^*sm13*^*+*^ transition state progenitor cells that commit to a *ludp*^+^ tegumental fate. If this were the case, we would anticipate that loss of *klf4*^+^ would result in increased birth of *ludp*^+^*sm13*^+^ transition state cells, yet not comprise the total number of new cells added to the tegument. To test this hypothesis, we knocked down *klf4* and performed an EdU pulse-chase experiment [11]. Specifically, we pulse-labeled neoblasts in control (RNAi) and *klf4* (RNAi) parasites with EdU and monitored the production of EdU^+^ tegumental cells and *ludp*^+^*sm13*^+^ tegument progenitors following a 7-day chase period. As anticipated, we found *klf4* RNAi led to no change in the percentage of newly produced tegument cells (Figs 6A and 6B). However, we found 52% of *sm13*^+^EdU^+^ cells expressed *ludp* in *klf4* (RNAi) parasites, which is significantly higher than in control (RNAi) worms (Figs 6C and 6D). Taken together these data suggest that loss of the one TRL leads to an increase in flux through the opposing TRL, in turn altering the molecular make-up of the tegument.

## Discussion

**Fig 7:**
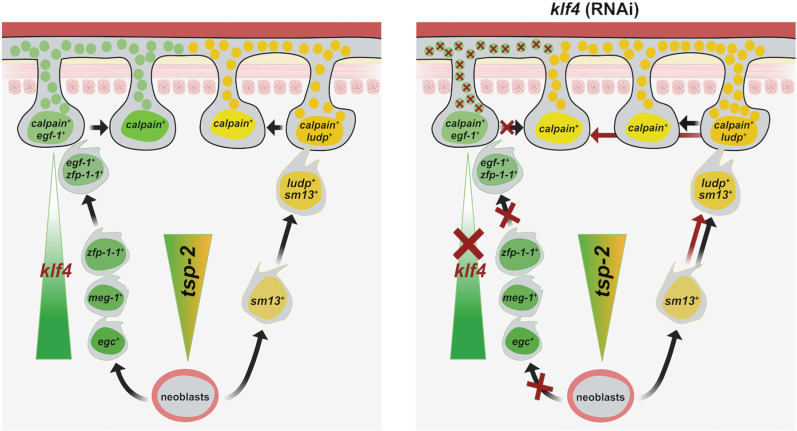
Model for the role of *klf4* in tegumental maintenance. Left: The tegument depends on pool of *tsp-2*^+^ tegument progenitor cells. Cells of the *egc*^+^/*meg-1*^+^/*zfp-1-1*^+^ TRL commit to transition state *egf-1*^+^ cells that fuse with the tegument, while *sm13*^*+*^ TRL cells commit to transition state *ludp*^+^ cells that likewise fuse with the tegument. Each TRL delivers a unique set of protein cargos to the tegumental syncytium (green or yellow circles). Right: In *klf4* (RNAi) worms, loss of *klf4* blunts the commitment of neoblasts to the *egc*^+^/*meg-1*^+^/*zfp-1-1*^+^ TRL resulting in an increase in the rate at which *sm13*^*+*^ lineage seeds new tegumental cell birth, thereby leading to alternation in the molecular composition of the tegumental syncytium.

Tegumental maintenance is crucial for schistosome longevity within their human host and depends on sustained turnover from a pool of *tsp-2*^+^ tegument progenitor cells [11]. Here, we reveal that tegumental renewal depends on two opposing *tsp-2*^*+*^ TRLs: one that expresses *sm13* and another that expresses *egc*/*meg-1*/*zfp-1-1*. We show that ablation of a Krüppel-like factor 4 homolog results in loss of the entire *egc*^+^/*meg-1*^+^/*zfp-1-1*^+^ TRL (Figs 3B, 3D and 3E) and loss of *egf-1*^+^ tegumental cells (Fig 4D). In parallel, loss of the *egc*^+^/*meg-1*^+^/*zfp-1-1*^+^ TRL is accompanied by an increase in the number of *sm13*^*+*^ and tegumental cells that express *ludp* (Figs 5C–5G). Surprisingly, while we observe loss of the entire *egc*^+^/*meg-1*^+^/*zfp-1-1*^+^ TRL following *klf4* RNAi treatment, there are no defects in the number of new tegumental cell being born (Figs 6A and 6B). Based on these data we propose a model whereby cells of the *egc*^*+*^/*meg-1*^+^/*zfp-1-1*^+^ TRL commit to *egf-1*^+^ cells that fuse with the tegument while *sm13*^*+*^ cells commit to *ludp*^+^ cells that likewise fuse with the tegument (Fig 7). This suggests that loss of *klf4* blunts the commitment of neoblasts to the *egc*^+^/*meg-1*^+^/*zfp-1-1*^+^ TRL leading to an increase in the rate at which *sm13*^*+*^ lineage seeds new tegumental cell birth. The cumulative effect of *klf4* depletion is an alternation in the molecular composition of the tegumental syncytium.

Why do the schistosomes appear to specify two molecularly distinct tegumental lineages? Despite being a syncytial tissue with continuous cytoplasm, classic ultrastructural studies of the tegument found various types of tegument-specific cytoplasmic inclusions which appeared to some extent be present in tegumental cells in a mutually-exclusive manner [10,18]. Thus, it is possible that these two lineages are destined to become two specific subtypes of tegumental cells possessing distinct molecular fingerprints and cellular compositions. If this is the case, then why do we only find that only a very small number of *calpain*^*+*^ tegumental cells that express markers indicating the presence of tegumental cell heterogeneity (i.e., *egf-1* and *ludp*) (Figs 4E and 5F)? Given that both *egf-1* and *ludp* are simultaneously expressed in both a subset of *calpain*^*+*^ tegmental cells and subsets of TRL^+^ cells, we argue that these are not stable, mature tegumental cells but rather cells that are in a transition state that have recently fused with the tegumental syncytium (Fig 7). Rather than tegumental cells actively expressing cell-type specific mRNAs to establish cellular heterogeneity, we favor a model in which molecularly distinct tegumental progenitors (i.e., *egc*^+^/*meg-1*^+^/*zfp-1-1*^+^ or *sm13*^*+*^ lineage cells) fuse with the tegument and bring with them a collection of specific proteins to execute distinct molecular functions. Indeed, this model is well-supported as proteins such as TSP-2 and SM13 are known to be present in the tegument [13,15], yet their mRNA are not observed in mature tegumental cell bodies [11]. Thus, it is likely that many proteins encoded by genes expressed in the respective TRLs make their way into the tegument. Understanding specific functions of these TRL-specific genes is expected to illuminate the roles for tegumental subpopulations in parasite biology.

Interestingly, one of the hallmarks of *klf4* RNAi is the loss of *zfp-1-1*^+^ cells combined with an apparent increase in flux through the *sm13*^+^ cell lineage. This result is somewhat paradoxical considering our previous work showing that *zfp-1-1* RNAi results in a loss of the *sm13*^+^ cell lineage [11]. If *zfp-1-1* RNAi results in loss of *sm13*^+^ cells, shouldn’t loss of *zfp-1-1*^+^ cells also result in a loss of *sm13*^+^ cells? scRNAseq helps resolve this apparent conflict: *zfp-1-1* is expressed not only in the late progenitors of the *klf4*^+^ lineage, but also in neoblast progeny and early *tsp-2*^+^ cells (Fig 1F). As such, loss of the *klf4*^+^ TRL results in only partial loss of *zfp-1-1* expression (S3G Fig); the *zfp-1-1* expression in neoblast progeny and early *tsp-2* progenitors remains intact (S3D Fig) allowing for the specification of the *sm13*^+^ TRL. This raises some interesting questions. In particular, does *zfp-1-1* regulate the same genes in neoblast progeny and in the *klf4*^+^ TRL? Furthermore, how is *zfp-1-1* expression regulated in each TRL such that it expressed only in the appropriate cell type? Clearly, future studies exploring the functions of *zfp-1-1* in these distinct cell compartments will bring important insights into these questions.

Tegument production and maintenance is a complex process [5] but pivotal for the success of the parasite in the blood. Here, we uncover *klf4* as a critical transcriptional regulator for maintaining heterogeneity within tegumental cell pool. Although the purpose of this tegumental heterogeneity is unclear, the development of robust experimental models that allow for the ablation of specific TRLs in bloodstream schistosomes is anticipated to unravel the functions of the various tegumental cell types. Such studies could reveal the molecular programs that mediate schistosome long-term survival and immune evasion and suggest new therapeutic interventions.

## Materials and methods

### Ethics statement

All experiments were performed with male parasites to maximize the amount of somatic tissue present. Experiments with and care of vertebrate animals were performed in accordance with protocols approved by the Institutional Animal Care and Use Committee (IACUC) of University of Texas Southwestern Medical Center (approval APN: 2017-102092).

### Parasite acquisition and culture

Adult *S. mansoni* (6–7 weeks post-infection) were obtained from infected female mice by hepatic portal vein perfusion with 37°C DMEM (Sigma-Aldrich, St. Louis, MO) plus 10% Serum (either Fetal Calf Serum or Horse Serum) and heparin. Parasites were cultured as previously described [12].

### RNA interference

20 freshly perfused male parasites were placed into 6-well plates and cultured in 10mL in Basch Media 169 supplemented with 30 μg/mL dsRNA for 17 days. dsRNA was replaced with fresh media on Day 0, 1, 2, 5, 8, 11. On day 14, the worms were given fresh media. On day 17, the worms were pulsed with 10 µM EdU for 4 hours before being fixed as previously described [19]; for EdU pulse-chase experiments, the worms were pulsed with 10 µM EdU for 4 hr after which the media was changed, the worms were fixed on day 24 following a 7 day chasing period. As a negative control for RNAi experiments, we used a non-specific dsRNA containing two bacterial genes [20]. cDNAs used for RNAi and *in situ* hybridization analyses were cloned as previously described [20]; To generate dsRNA, a 606 bp *klf4* PCR product was used as the template for *in vitro* transcription in a 100 µL reaction containing 59 µL DEPC-treated water, 20 µL 100mM mix of rNTPs (Promega, E6000), 10 µL high-yield transcription buffer (0.4 M Tris pH 8.0, 0.1 M MgCl_2_, 20 mM spermidine, 0.1 M DTT), 1 µL thermostable inorganic pyrophosphatase (New England Biolabs, M0361L) and 5 µL HIS-Tagged T7 RNA polymerase. The reaction was incubated at 37°C at least 4hrs, then treated with 5 µL RQ1 RNase-free DNase (Promega, M6101). Synthesized RNA was then melted by heating at 95°C, 75°C and 55°C each for 3 min, to allow annealing of the sense and antisense strands into dsRNA; oligonucleotide primer sequences and the DNA template sequence for the *klf4* dsRNA are listed in S5 Dataset.

### *In vitro* EdU pulse chase experiment

Freshly perfused adult male parasites, cultured in Basch Media 169, were pulsed with 10 µM EdU for 4 hours. After the pulse, the media was replaced, and the worms were either fixed immediately (D0) or cultured as above and fixed after 1, 2, 3, and 5 days of chase period. At least four worms were imaged for cell quantification at each time point.

### qPCR and RNAseq analysis

Whole parasites were collected and homogenized in Trizol, RNA extraction, cDNA preparation and qPCR were performed as previously described [21]. Oligonucleotide primer sequences used for qPCR are listed in S5 Dataset. For RNAseq analysis, three biological replicates were performed for control treatment and knockdown condition. The samples were prepared by Illumina TruSeq stranded mRNA library kit. All 6 samples were sequenced with one flow cell on Illumina NextSeq 550 sequencer with 75 bp read lengths. Reads were mapped with STAR (v2.7.10a)[22] and *S. mansoni* genome sequence (v7) and GTF files used for mapping were acquired from Wormbase Parasite [23]. Differential gene expression were performed with DESeq2 (version 1.30.1) [24] R(4.0.3). Raw and processed data have been deposited in NCBI (GSE268037). Volcano plots were made with plotting log2 fold change expression and -log10 (*P*adj) of differential expressed genes (*P*adj < 0.05) in GraphPad Prism.

### Fluorescence-activated cell sorting

FACS sorting was performed as previously described [14] with minor modifications. After RNAi experiments, 100 male worms from each group were suspended in a 0.5% solution of Trypsin/EDTA (Sigma T4174) in PBS before rinsing in PBS for triple times. The worms were then triturated for approximately 10 minutes at room temperature until the solution became turbid and no large pieces of worms were left. The trypsin was inactivated by adding an equal volume of Basch media. The dissociated worms were then centrifuged at 500 g for 10 min at 4°C. Next the worms were resuspended in 1 mL of Basch medium followed by filtering through 100 μm cell strainer to remove big chunks. The filtered collections were treated with 10 μL of RQ1 DNase (Promega M6101) and incubated for 10 minutes at RT. After adding 3mL PBS, the dissociated worms were centrifuged again at 500 g for 10 minutes at 4°C. The cells were then resuspended in 1mL of staining media (0.2% BSA, 2mM EDTA in PBS, pH 7.40) and incubated in Hoechst 33342 (18 μg/mL) (Sigma B2261) for 30min at RT in the dark. The worms were centrifuged once again at 500 g for 10 minutes at 4°C. Worms were then resuspended in 1 mL of staining media containing Hoechst 33342 (18 μg/mL) and propidium iodide (1 μg/mL) (Sigma-Aldrich P4170) and then filtered through a 100 μm cell strainer into a 12x75mm FACS tube prior to sorting. Filtered cells were then sorted on a FACSAria II custom (BD Biosystems) with 305/405/488/561/633nm lasers. Live single cells (PI negative, singlet by comparing forward scatter height to forward scatter width) were sorted using a 100 μm nozzle and cells were sorted into sorting media (0.2% BSA in PBS, pH 7.4). A Hoechst threshold was applied to exclude debris and improve the efficiency of sorting.

### Single-cell RNA sequencing

For sub-clustering the tegument related cell populations from the adult scRNAseq dataset (GSE146736_adult_scseq_seurat), we use the subset() command to make a new Seurat object that included neoblasts, *dmrt1*^+^ neoblasts, neoblast progeny, *hes2*^+^, early *tsp-2*^+^, *sm13*^+^, *egc*^+^, *meg-1*^+^, *zfp-1-1*^+^, tegument1 and tegument2 clusters, and ranRunUMAP (reduction = “pca”, dims = 1:50, n.neighbors =50, min.dist=50), FindNeighbors (reduction = “pca”, dims = 1:50), FindClusters (resolution = 0.5), then we got a final UMAP with 12 clusters.

FACS-sorted cells were centrifuged again at 500 g for 10 minutes at 4°C then resuspended in 0.2% BSA in PBS. Libraries were created using a Chromium Controller (10x Genomics) according to manufacturer guidelines and sequenced using a NextSeq 500 (illumina). Sequencing data was processed and mapped to the *Schistosoma mansoni* genome (v7) using Cell Ranger (10x Genomics). Unfiltered data from Cell Ranger was imported into Seurat (v4.3) [25, 26] and cells were filtered as follows: nFeature_RNA > 200 & nFeature_RNA<4000 & nCount_RNA > 1000 & nCount_RNA < 20000 & percent.mt < 5 (Mitochondrial genes were identified as those with the prefix “Smp_9”). Control (RNAi) and *klf4* (RNAi) datasets were normalized (NormalizeData) and variable features were identified (FindVariableFeatures, selection.method = “vst”, nfeatures =2000). From here, integration anchors were identified (FindIntegrationAnchors, dims 1:60), the data was integrated (IntegrateData, dims = 1:60, features.to.integrate = all.genes), and scaled (ScaleData). We then ran RunPCA, RunUMAP (reduction = “pca”, dims = 1:60, n.neighbors =30), FindNeighbors (reduction = “pca”, dims = 1:60), FindClusters (resolution = 4). After merging we were left with a final map of 65 clusters of 12910 cells. To identify the clusters of tissue distribution on the UMAP, we used FindAllMarkers (avg_log2FC>1) command to identify marker genes of each cluster (S3 Dataset), and validated by manually inspecting tissue markers, including *egc*^*+*^ (Smp_314170), *meg-1*^*+*^ (Smp_122630), *zfp-1-1*^*+*^ (Smp_049580), early *tsp-2*^*+*^ (Smp_335630), *sm13*^*+*^ (Smp_195190), tegument (*calpain,* Smp_214190), tegument2 (Smp_056460), neoblast progeny (Smp_171720*; hes2,* Smp_132810), neoblasts (*nanos2*, Smp_051920*; eled*, Smp_041540), neurons (*7b2,* Smp_073270), flame cells (*sialidase*, Smp_335600), muscle (*tpm2,* Smp_031770), parenchyma (*tgfbi,* Smp_212710) and gut (*hnf4*, Smp_174700; *ctsb*, Smp_103610) [14]. Then we collapsed all tissue-specific clusters into a single cluster and assigned them same color and name, generated the UMAP of merged datasets (S3A Fig) and the marker list of each labeled cluster (S4 Dataset). The dot plots for Figs 3D, S3F and S5A were generated using the DotPlot() function in Seurat v4.3 with the all down-regulated genes and up-regulated genes following *klf4* RNAi (S2 Dataset). The size of the dot corresponds to the percentage of the cells in the cluster (indicated on the vertical axis) that express the given gene (indicated on the horizontal axis), whereas the color of the dot indicates the average expression level of the gene in the cluster. To identify differential expression genes in same cell types between control (RNAi) and *klf4* (RNAi) worms, since *egc*^*+*^ and *meg-1*^*+*^& *zfp-1-1*^*+*^ populations were too small to perform differential analysis, we combined the *egc*^*+*^*, meg-1*^*+*^& *zfp-1-1*^*+*^*,* tegument and tegument2 clusters into a single “*klf4*^+^ TRL” cluster and combined the early *tsp-2*^+^ and *sm13*^+^ clusters into a single “*sm13*^+^ TRL” cluster, then using the FindMarkers() command to identify differential expressed genes of these clusters (S1 Dataset). Raw and processed data have been deposited in NCBI (GSE268036).

### Parasite labeling and imaging

Colorimetric and fluorescence *in situ* hybridization detections were performed as previously described [12,19] with the following modification. To improve signal-to-noise for colorimetric *in situ* hybridization, all probes were used at 10 ng/mL in hybridization buffer. For FISH, we prolonged the TSA (Tyramide Signal Amplification) reaction time to 20 or 40min with 50 ng/mL probe or 10ng/mL probe, respectively. EdU detection was performed as previously described [19]. All fluorescently labeled parasites were counterstained with DAPI (1 μg/mL), cleared in 80% glycerol, and mounted on slides with Vectashield (Vector Laboratories).

Confocal imaging of fluorescently labeled samples was performed on either a Zeiss LSM900 or a Nikon A1 Laser Scanning Confocal Microscope. All images were captured from the mid-trunk region of male parasites. To perform cell counts, cells were manually counted in maximum intensity projections derived from confocal stacks. In cases where we determined the number of cells in a particular region of the parasite (e.g., tegument), we collected confocal stacks and normalized the number of cells by total volume of the stack in μm^3^. In cases where we determined the total number of labeled foci throughout the entire depth of the parasite (e.g., *tsp-2*^*+*^ counts), we collected confocal stacks and normalized the number of cells to the length of the parasite in the imaged region in mm. In cases where we determined the percentage of each interactive cell populations, we captured confocal stacks from the middle trunk of the worms, counted the number of cells for each population of interest, and assessed co-localization between different populations. The Venn diagrams were generated by https://academo.org/demos/venn-diagram-generator/ using the percentage of each interactive cell populations, calculated from cell counts. Brightfield images were acquired on a Zeiss AxioZoom V16 equipped with a transmitted light base and a Zeiss AxioCam 105 Color camera.

### Statistical analysis

GraphPad Prism software processed and presented the data as the mean with standard deviation (mean ± SD). All pairwise comparisons were analyzed using Welch’s t-test except for those in Figs 3G, 4B, 6B and S3H, where unpaired t-tests were performed. Paired t-tests were performed in Figs 3H, 4C and multiple paired t-tests were performed in Figs 5B, S3G and S4A.

## Supporting information

S1 Fig scRNAseq atlas of the adult schistosome reveals two tegument related lineages (TRLs). (A) UMAP plot showing the tegumental markers (Smp_214190 *calpain,* Smp_153390 *npp-5,* Smp_077720 *annexin* and Smp_105410 *gtp-4*) from scRNAseq atlas of male adult *S. mansoni*. (B) UMAP plot showing sub-clustering within the tegument-associated populations of adult schistosomes. (C-P) EdU pulse-chase experiment examining the kinetics of EdU incorporation into TRLs, including *egc*^+^ cells (C, D), *meg-1*^+^ cells (E, F), *zfp-1-1*^+^ cells (G, H), *klf4*^+^ cells (I, J), *tsp-2*^+^ cells (K, L), *sm13*^+^ cells (M, N) and tegumental cells (O-P). FISH for *egc* (C), *meg-1* (E), *zfp-1-1* (G), *klf4* (I), *tsp-2* (K), *sm13* (M) and tegument (O) markers with EdU detection at D0, D1, D2, D3 and D5 following an EdU pulse. Arrows represent EdU^+^ cells. Scale bars: 10 µm. Quantification of the percentage of EdU^+^ cells in *egc*^+^ (D), *meg-1*^+^ (F), *zfp-1-1*^+^ (H), *klf4*^+^ (J), *tsp-2*^+^ (L), *sm13*^+^ (N) and tegument^+^ (P) cells. Data are presented as mean ± SD.
(TIFF)

S2 Fig *Sm*KLF4 belongs to the KLF4 family. (A) Top: Protein sequence alignment of *Sm*KLF4 with KLF4 from *Danio rerio* (NP_001106955.1), *Xenopus tropicalis* (NP_001017280.1), *Mus musculus* (NP_034767.2) and *Homo sapiens* (NP_001300981.1). *Sm*KLF4 is 547 amino acids in length and contains a 9AA transactivation domain (TAD) [27] located at position AA73-81 (highlighted in pink rectangle). The acidic amino acids glutamine (E) and aspartate (D) in humans are indicated with pink triangles, filled triangle represents identical residues in the *S. mansoni* protein and empty triangles indicates non-identical residues. At the C-terminus, *Sm*KLF4 features an 81AA highly conserved region containing three C2H2 zinc finger domains. The amino acid sequence below is highlighted in filled yellow rectangles, with cystine and histidine residues highlighted in green. Fifteen highly conserved basic residues (KKKRKr/kRRRK RRRKK) are marked with red stars, with only one non-identical residues labelled with an empty star, nine conserved residues crucial for DNA binding specificity are highlighted with filled green circles. Bottom: Protein sequence alignment of *Sm*KLF4 with its paralogs from *S. mansoni*. (B) WISH results showing expression pattern of *egc, meg-1, zfp-1-1, tsp-2* and *sm13*. Scale bar, 100 µm. (C) *klf4* (246 cells) has no expression in tegument progenitor *sm13*^+^ cells (250 cells) (0/250, n=6 parasites). Scale bar, 10 µm.
(TIFF)

S3 Fig *Klf4* is required for *egc* ^*+*^ */meg-1* ^*+*^ */zfp-1-1* ^*+*^ TRL. (A) UMAP plot of all clusters and their predicted cellular identity from *klf4* RNAi scRNAseq analysis. (B-E) UMAP plot shows expression of (B) *egc*, (C) *meg-1*, (D) *zfp-1-1* and (E) *tsp-2* in control (RNAi) and *klf4* (RNAi) worms. (F) A dot-plot summarizing the expression of all bulk RNAseq down-regulated DEGs in the *klf4* RNAi-mediated scRNAseq profile. Cluster populations are on the vertical axis and gene IDs are on the horizontal axis. Expression levels are colored by gene expression (blue = low, red = high). Percentage of cells in the cluster expressing the gene is indicated by the size of the circle. (G) qPCR detection of expression of *klf4* (n=10 experiments)*, egc* (n=8 experiments)*, meg-1* (n=7 experiments) and *zfp-1-1* (n=9 experiments) following *klf4* RNAi. (H) Quantification of the number of EdU^+^ cells per mm of worms. Control (RNAi) n= 35, *klf4* (RNAi) n=34. Data are presented as mean ± SD. Multiple paired t-tests and an unpaired t-test were performed in Panel G and H, respectively.
(TIFF)

S4 Fig *egc* ^+^/*meg-1* ^+^/*zfp-1-1* ^+^ TRL is required for producing tegumental subpopulations. (A) qPCR detection of expression of *egf-1* (n=5 experiments), *meg-10* (n=6 experiments), *M13 peptidase* (n=4 experiments) and *rpn-2* (n=3 experiments) following *klf4* RNAi. Data are presented as mean ± SD and multiple paired t-tests were performed. (B) Left, FISH results confirm a loss of *M13 peptidase*^+^ and *rpn-2*^+^ cells (highlight with magenta dot in (Fig 3C)) following *klf4* RNAi. Numbers at bottom left represent the fraction of parasites displaying the observed phenotype. Right, Double FISH showing expression of *M13 peptidase* and *rpn-2* relative to the *calpain*^+^ and *zfp-1-1*^+^ cells, respectively (indicated in arrows). The Venn diagram in upper right shows the percentage of *M13 peptidase*^+^ cells (114/310 cells are *calpain*^+^, n=16 parasites; 45/71 cells are *zfp-1-1*^+^, n=6 parasites) and *rpn-2*^+^ cells (197/637 cells are *calpain*^+^, n=10 parasites; 212/384 cells are *zfp-1-1*^+^, n=9 parasites) in *calpain*^+^ and *zfp-1-1*^+^ cells. Scale bar, 100 µm. (C) WISH showing expression pattern of *egf-1*, *meg-10*, *M13 peptidase* and *rpn-2*. Scale bar, 100 µm. (D) UMAP plot shows expression pattern of *egf-1*, *meg-10*, *M13 peptidase* and *rpn-2* on the established adult scRNAseq atlas*.*
(TIFF)

S5 Fig Knockdown of *klf4* increases heterogeneity in *sm13* ^*+*^ progenitor cells. (A) Dot-plot summarizing the expression of the up-regulated DEGs from the bulk RNAseq analysis on the *klf4* RNAi scRNAseq profile. The genes enriched in the *sm13*^+^ cell population are indicated in star and dashed rectangle (Left), with the corresponding UMAP plot from scRNAseq of male adult schistosome (Right). (B) FISH results showing an increase in the number of *ly6e*^+^ cells following *klf4* RNAi (left); double FISH showing expression of *sm13* relative to the *ly6e*^+^ cells (right), white arrows indicate the *sm13*^+^*ly6e*^+^ cells and red arrows indicate the *sm13*^-^*ly6e*^+^ cells. Venn diagram in upper right shows the percentage of *sm13*^*+*^ cells expressing *ly6e*. Scale bar, 100 µm. (C) Quantification of the number of *ly6e*^+^ cells per mm of worms. Control (RNAi) n=18, *klf4* (RNAi) n=18. (D) Quantification of percentage of *sm13*^*+*^ cells expressing *ly6e*. Control (RNAi) n=16, *klf4* (RNAi) n=15. (E) Double FISH showing expression of *calpain* relative to the *ly6e*^+^ cells, pink arrows indicate the *calpain*^*+*^*ly6e*^+^ cells. Venn diagram in upper right shows the percentage of *calpain*^*+*^ cells expressing *ly6e*. (F) Quantification of the percentage of *calpain*^*+*^ cells expressing *ly6e*. Control (RNAi) n=5, *klf4* (RNAi) n=7. (G) FISH results showing an increase in the number of *litaf*^+^ cells following *klf4* RNAi (left); double FISH showing expression of *sm13* relative to the *litaf*^+^ cells (right), white arrows indicate the *sm13*^+^*litaf*^+^ cells, red arrows indicate the *sm13*^-^*litaf*^+^ cells. Venn diagram in upper right shows the percentage of *sm13*^*+*^ cells expressing *litaf*. Scale bar, 100 µm. (H) Quantification of the number of *litaf*^+^ cells per mm of worms. Control (RNAi) n=19, *klf4* (RNAi) n=14. (I) Quantification of percentage of *sm13*^*+*^ cells expressing *litaf*. Control (RNAi) n=14, *klf4* (RNAi) n=10. (J) Double FISH showing expression of *calpain* relative to the *litaf*^+^ cells, and the percentage of *calpain*^+^ cells expressing *litaf* gene was quantified that shown in Venn diagram in upper right. (K) Quantification of percentage of *calpain*^*+*^ cells expressing *litaf*. Control (RNAi) n=10, *klf4* (RNAi) n=9. Scale bar, 10 µm. Data are presented as mean ± SD. Welch’s t-tests were performed in Panel C, D, F, H, I and K.
(TIFF)

S1 Dataset Differential expression analysis within single cell clusters from single-cell RNA sequencing (scRNAseq) studies comparing control (RNAi) and *klf4* (RNAi) treatments. (XLSX)

S2 Dataset Differentially expressed genes (DEGs) from bulk RNAseq analysis following *klf4* RNAi. (XLSX)

S3 Dataset List of genes significantly enriched in all clusters (65 clusters in total) from scRNAseq analysis. (XLSX)

S4 Dataset List of genes significantly enriched in the same cell types from scRNAseq analysis. (XLSX)

S5 Dataset List of oligos and abbreviations used in this study. (XLSX)
