# Supplementary figures and images for: A Krüppel-like factor establishes cellular heterogeneity during schistosome tegumental maintenance
Source: PLoS Pathog. 2025 Mar 28;21(3):e1013002. doi: 10.1371/journal.ppat.1013002 (PMC11978072; doi:10.1371/journal.ppat.1013002)

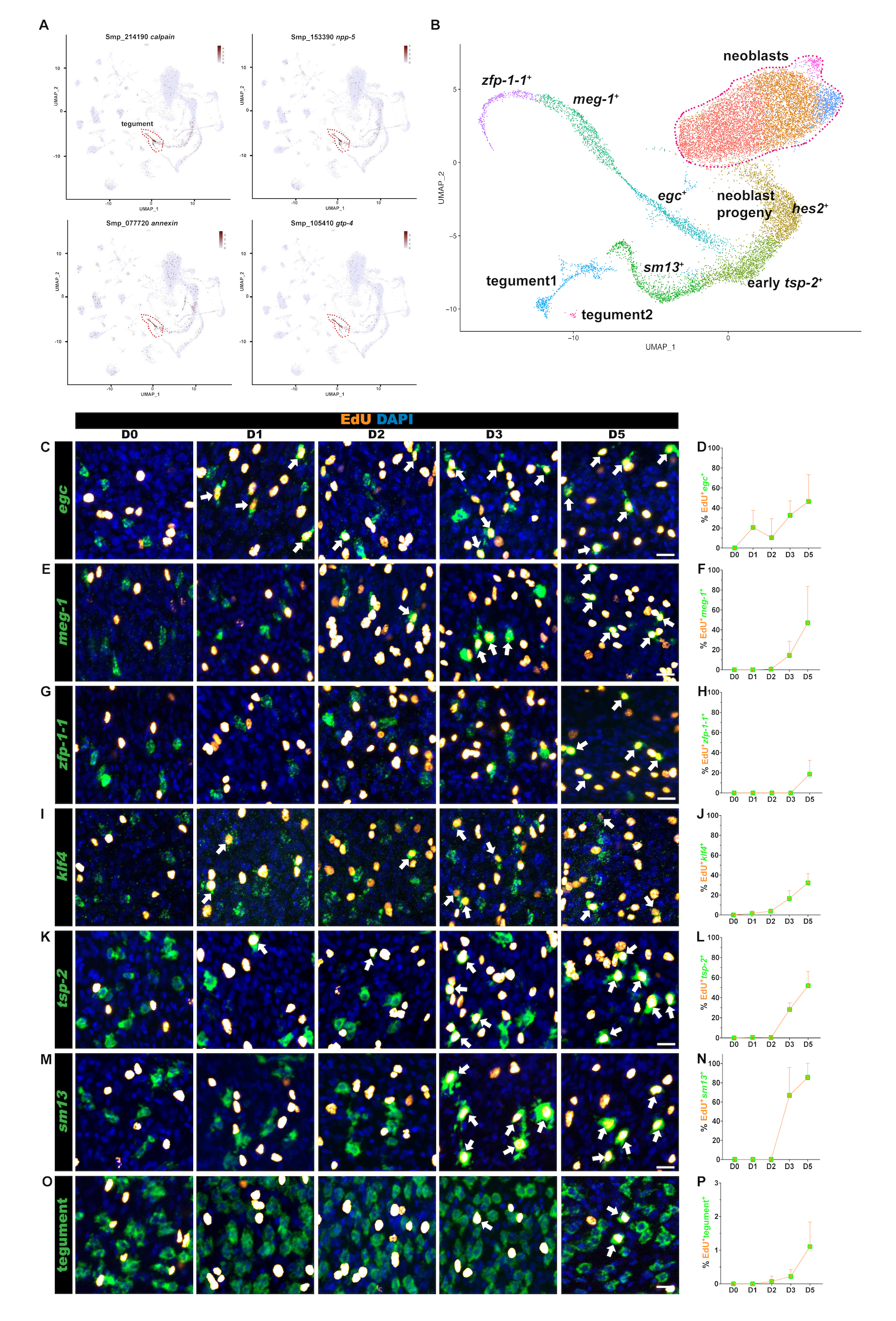

Supplement: S1 Fig — (A) UMAP plot showing the tegumental markers (Smp_214190 calpain, Smp_153390 npp-5, Smp_077720 annexin and Smp_105410 gtp-4) from scRNAseq atlas of male adult S. mansoni. (B) UMAP plot showing sub-clustering within the tegument-associated populations of adult schistosomes. (C-P) EdU pulse-chase experiment examining the kinetics of EdU incorporation into TRLs, including egc+ cells (C, D), meg-1+ cells (E, F), zfp-1-1+ cells (G, H), klf4+ cells (I, J), tsp-2+ cells (K, L), sm13+ cells (M, N) and tegumental cells (O-P). FISH for egc (C), meg-1 (E), zfp-1-1 (G), klf4 (I), tsp-2 (K), sm13 (M) and tegument (O) markers with EdU detection at D0, D1, D2, D3 and D5 following an EdU pulse. Arrows represent EdU+ cells. Scale bars: 10 µm. Quantification of the percentage of EdU+ cells in egc+ (D), meg-1+ (F), zfp-1-1+ (H), klf4+ (J), tsp-2+ (L), sm13+ (N) and tegument+ (P) cells. Data are presented as mean ± SD. (TIFF) [file ppat.1013002.s001.tiff]

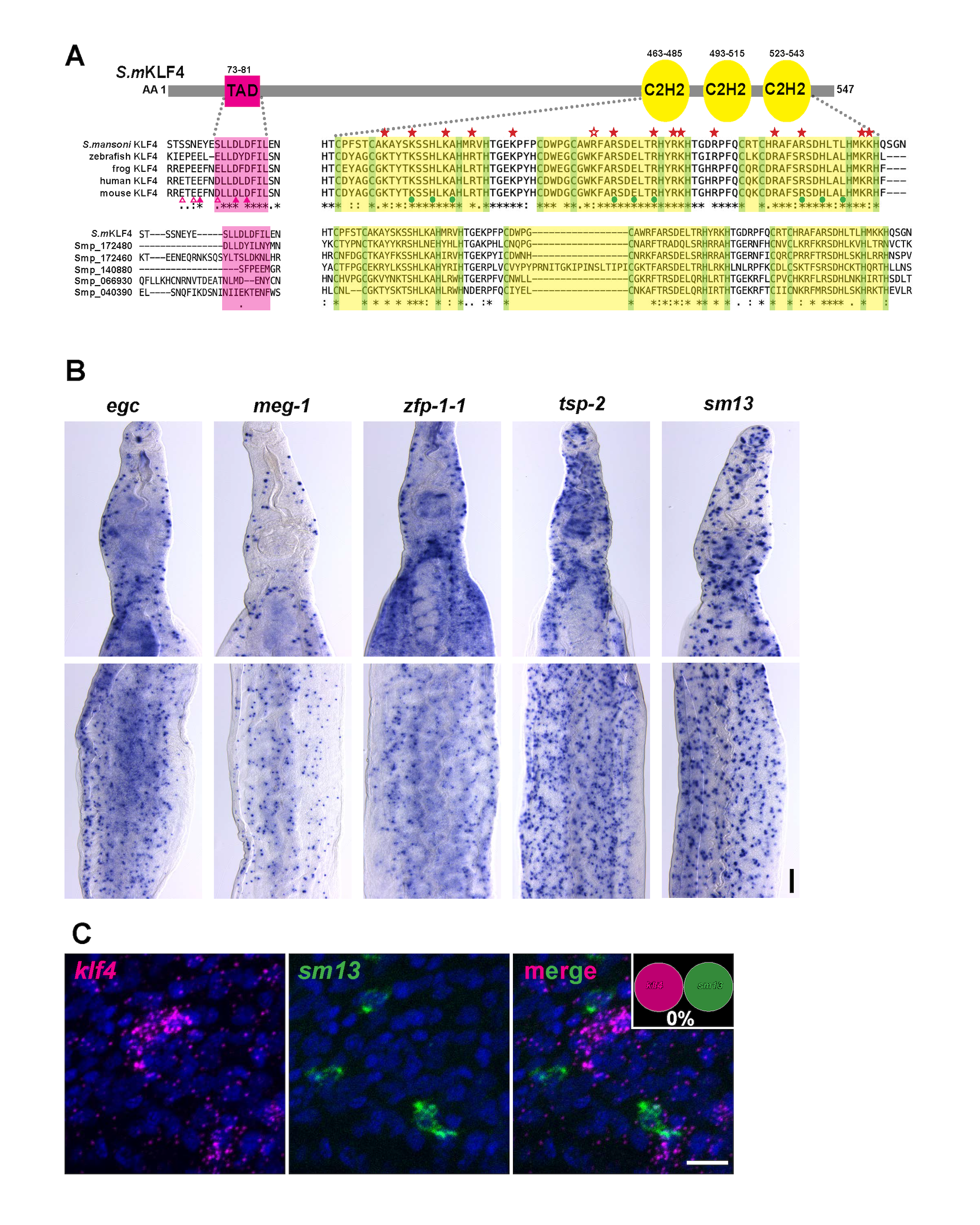

Supplement: S2 Fig — (A) Top: Protein sequence alignment of SmKLF4 with KLF4 from Danio rerio (NP_001106955.1), Xenopus tropicalis (NP_001017280.1), Mus musculus (NP_034767.2) and Homo sapiens (NP_001300981.1). SmKLF4 is 547 amino acids in length and contains a 9AA transactivation domain (TAD) [27] located at position AA73-81 (highlighted in pink rectangle). The acidic amino acids glutamine (E) and aspartate (D) in humans are indicated with pink triangles, filled triangle represents identical residues in the S. mansoni protein and empty triangles indicates non-identical residues. At the C-terminus, SmKLF4 features an 81AA highly conserved region containing three C2H2 zinc finger domains. The amino acid sequence below is highlighted in filled yellow rectangles, with cystine and histidine residues highlighted in green. Fifteen highly conserved basic residues (KKKRKr/kRRRK RRRKK) are marked with red stars, with only one non-identical residues labelled with an empty star, nine conserved residues crucial for DNA binding specificity are highlighted with filled green circles. Bottom: Protein sequence alignment of SmKLF4 with its paralogs from S. mansoni. (B) WISH results showing expression pattern of egc, meg-1, zfp-1-1, tsp-2 and sm13. Scale bar, 100 µm. (C) klf4 (246 cells) has no expression in tegument progenitor sm13+ cells (250 cells) (0/250, n=6 parasites). Scale bar, 10 µm. (TIFF) [file ppat.1013002.s002.tiff]

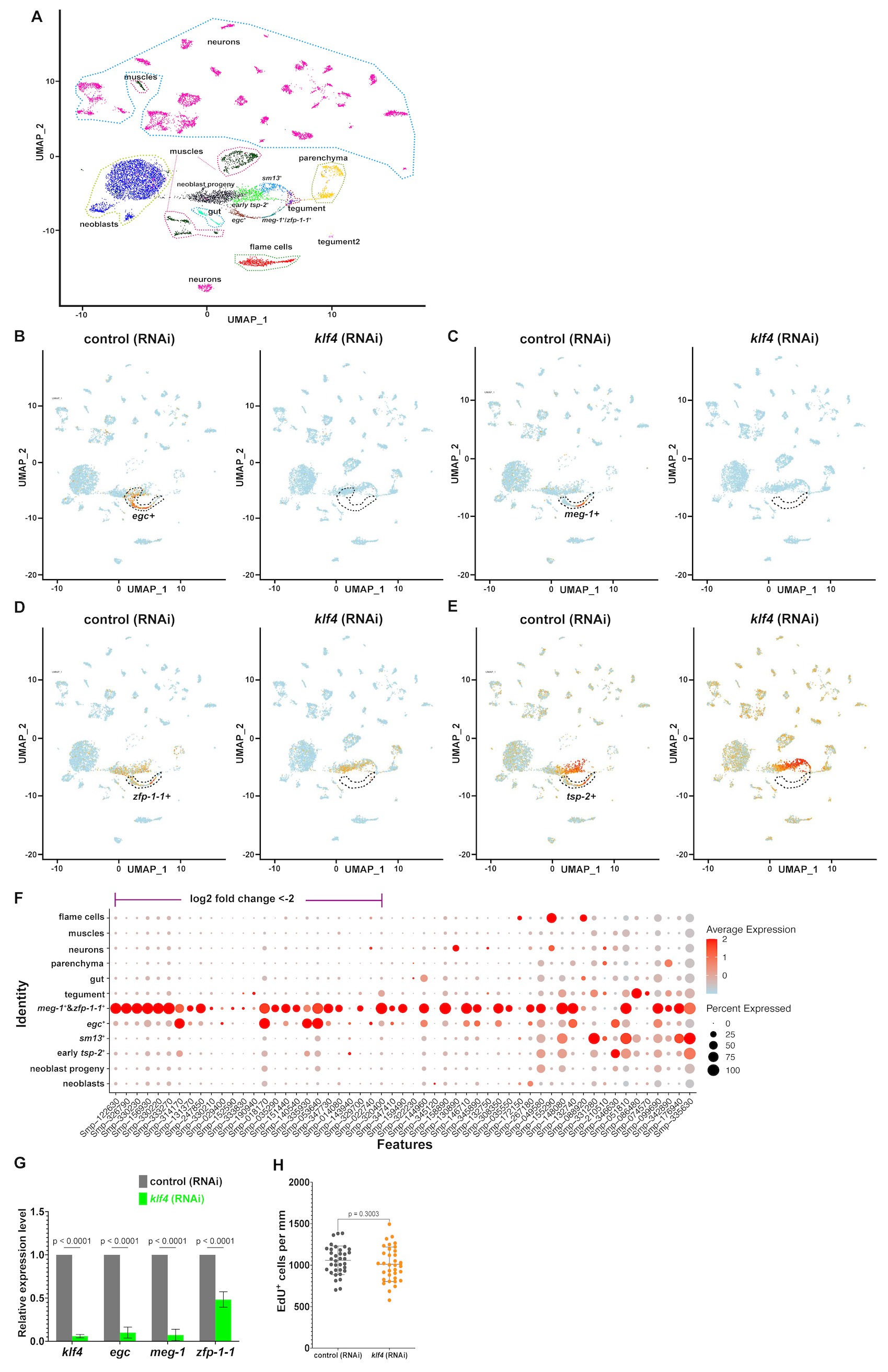

Supplement: S3 Fig — (A) UMAP plot of all clusters and their predicted cellular identity from klf4 RNAi scRNAseq analysis. (B-E) UMAP plot shows expression of (B) egc, (C) meg-1, (D) zfp-1-1 and (E) tsp-2 in control (RNAi) and klf4 (RNAi) worms. (F) A dot-plot summarizing the expression of all bulk RNAseq down-regulated DEGs in the klf4 RNAi-mediated scRNAseq profile. Cluster populations are on the vertical axis and gene IDs are on the horizontal axis. Expression levels are colored by gene expression (blue = low, red = high). Percentage of cells in the cluster expressing the gene is indicated by the size of the circle. (G) qPCR detection of expression of klf4 (n=10 experiments), egc (n=8 experiments), meg-1 (n=7 experiments) and zfp-1-1 (n=9 experiments) following klf4 RNAi. (H) Quantification of the number of EdU+ cells per mm of worms. Control (RNAi) n= 35, klf4 (RNAi) n=34. Data are presented as mean ± SD. Multiple paired t-tests and an unpaired t-test were performed in Panel G and H, respectively. (TIFF) [file ppat.1013002.s003.tiff]

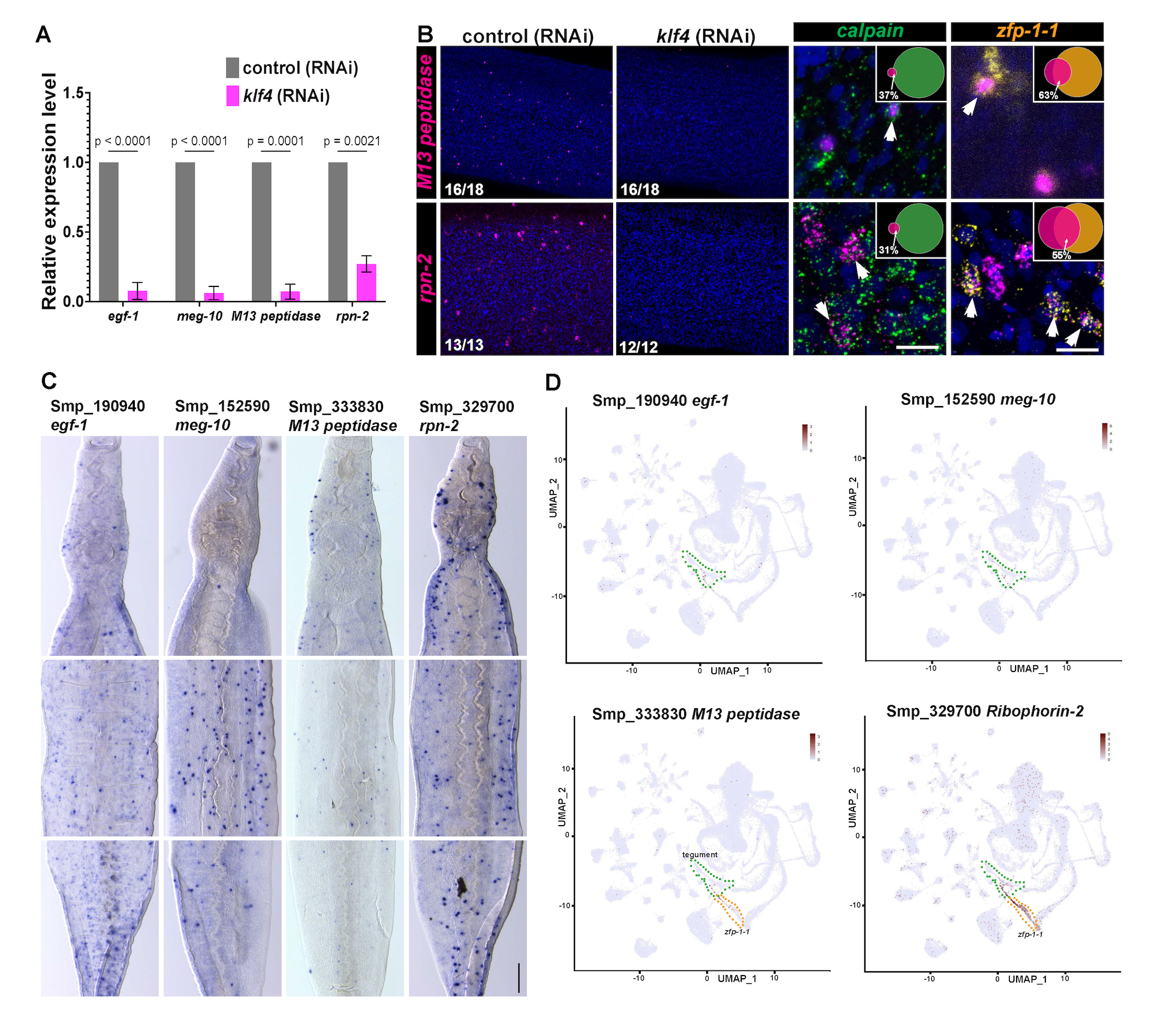

Supplement: S4 Fig — (A) qPCR detection of expression of egf-1 (n=5 experiments), meg-10 (n=6 experiments), M13 peptidase (n=4 experiments) and rpn-2 (n=3 experiments) following klf4 RNAi. Data are presented as mean ± SD and multiple paired t-tests were performed. (B) Left, FISH results confirm a loss of M13 peptidase+ and rpn-2+ cells (highlight with magenta dot in (Fig 3C)) following klf4 RNAi. Numbers at bottom left represent the fraction of parasites displaying the observed phenotype. Right, Double FISH showing expression of M13 peptidase and rpn-2 relative to the calpain+ and zfp-1-1+ cells, respectively (indicated in arrows). The Venn diagram in upper right shows the percentage of M13 peptidase+ cells (114/310 cells are calpain+, n=16 parasites; 45/71 cells are zfp-1-1+, n=6 parasites) and rpn-2+ cells (197/637 cells are calpain+, n=10 parasites; 212/384 cells are zfp-1-1+, n=9 parasites) in calpain+ and zfp-1-1+ cells. Scale bar, 100 µm. (C) WISH showing expression pattern of egf-1, meg-10, M13 peptidase and rpn-2. Scale bar, 100 µm. (D) UMAP plot shows expression pattern of egf-1, meg-10, M13 peptidase and rpn-2 on the established adult scRNAseq atlas. (TIFF) [file ppat.1013002.s004.tiff]

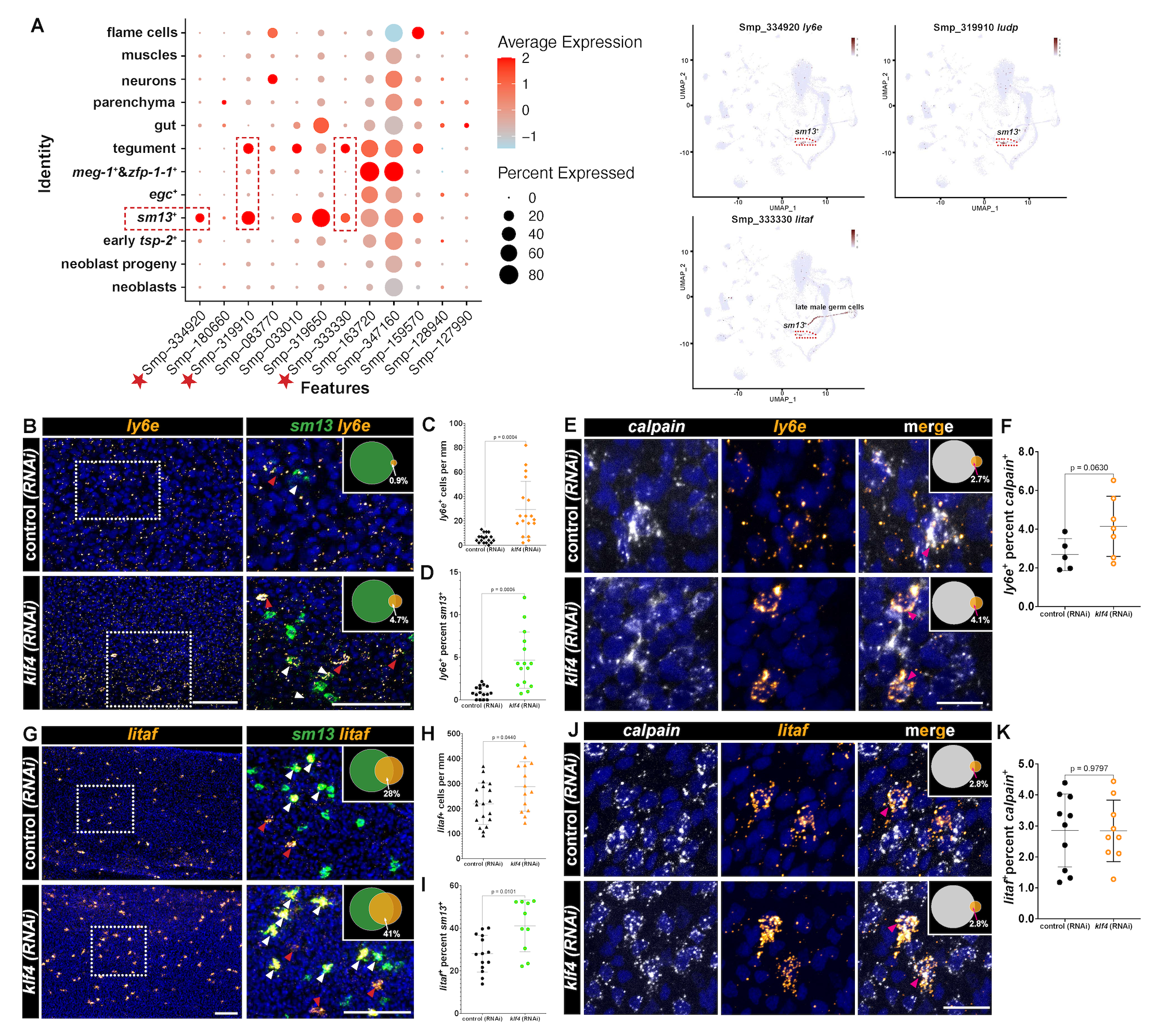

Supplement: S5 Fig — (A) Dot-plot summarizing the expression of the up-regulated DEGs from the bulk RNAseq analysis on the klf4 RNAi scRNAseq profile. The genes enriched in the sm13+ cell population are indicated in star and dashed rectangle (Left), with the corresponding UMAP plot from scRNAseq of male adult schistosome (Right). (B) FISH results showing an increase in the number of ly6e+ cells following klf4 RNAi (left); double FISH showing expression of sm13 relative to the ly6e+ cells (right), white arrows indicate the sm13+ly6e+ cells and red arrows indicate the sm13-ly6e+ cells. Venn diagram in upper right shows the percentage of sm13+ cells expressing ly6e. Scale bar, 100 µm. (C) Quantification of the number of ly6e+ cells per mm of worms. Control (RNAi) n=18, klf4 (RNAi) n=18. (D) Quantification of percentage of sm13+ cells expressing ly6e. Control (RNAi) n=16, klf4 (RNAi) n=15. (E) Double FISH showing expression of calpain relative to the ly6e+ cells, pink arrows indicate the calpain+ly6e+ cells. Venn diagram in upper right shows the percentage of calpain+ cells expressing ly6e. (F) Quantification of the percentage of calpain+ cells expressing ly6e. Control (RNAi) n=5, klf4 (RNAi) n=7. (G) FISH results showing an increase in the number of litaf+ cells following klf4 RNAi (left); double FISH showing expression of sm13 relative to the litaf+ cells (right), white arrows indicate the sm13+litaf+ cells, red arrows indicate the sm13-litaf+ cells. Venn diagram in upper right shows the percentage of sm13+ cells expressing litaf. Scale bar, 100 µm. (H) Quantification of the number of litaf+ cells per mm of worms. Control (RNAi) n=19, klf4 (RNAi) n=14. (I) Quantification of percentage of sm13+ cells expressing litaf. Control (RNAi) n=14, klf4 (RNAi) n=10. (J) Double FISH showing expression of calpain relative to the litaf+ cells, and the percentage of calpain+ cells expressing litaf gene was quantified that shown in Venn diagram in upper right. (K) Quantification of percentage of calpain+ [file ppat.1013002.s005.tiff]
